# Improving Payload Capacity and Anti-Tumor Efficacy of Mesenchymal Stem Cells Using TAT Peptide Functionalized Polymeric Nanoparticles

**DOI:** 10.3390/cancers11040491

**Published:** 2019-04-06

**Authors:** Gopikrishna Moku, Buddhadev Layek, Lana Trautman, Samuel Putnam, Jayanth Panyam, Swayam Prabha

**Affiliations:** 1 Department of Pharmaceutics, College of Pharmacy, University of Minnesota, Minneapolis, MN 55455, USA; gopikrishna.moku@gmail.com (G.M.); jpanyam@umn.edu (J.P.); 2 Department of Experimental and Clinical Pharmacology, College of Pharmacy, University of Minnesota, Minneapolis, MN 55455, USA; blayek@umn.edu; 3 Breck School, 123 Ottawa Ave N, Golden Valley, MN 55422, USA; traula@student.breckschool.org (L.T.); putnsa@student.breckschool.org (S.P.)

**Keywords:** mesenchymal stem cells (MSCs), TAT peptide, PLGA, paclitaxel, nano-engineered MSCs, orthotopic lung tumor model

## Abstract

Mesenchymal stem cells (MSCs) accumulate specifically in both primary tumors and metastases following systemic administration. However, the poor payload capacity of MSCs limits their use in small molecule drug delivery. To improve drug payload in MSCs, we explored polymeric nanoparticles that were functionalized with transactivator of transcription (TAT) peptide. Paclitaxel loaded poly(DL-lactide-co-glycolide) (PLGA) nanoparticles (15–16% *w*/*w* paclitaxel; diameter of 225 ± 7 nm; and zeta potential of −15 ± 4 mV) were fabricated by emulsion-solvent evaporation method, followed by TAT-conjugation to the surface of nanoparticles via maleimide-thiol chemistry. Our studies demonstrated that TAT functionalization improved the intracellular accumulation and retention of nanoparticles in MSCs. Further, nano-engineering of MSCs did not alter the migration and differentiation potential of MSCs. Treatment with nano-engineered MSCs resulted in significant (*p* < 0.05) inhibition of tumor growth and improved survival (*p* < 0.0001) in a mouse orthotopic model of lung cancer compared to that with free or nanoparticle encapsulated drug. In summary, our results demonstrated that MSCs engineered using TAT functionalized nanoparticles serve as an efficient carrier for tumor specific delivery of anticancer drugs, resulting in greatly improved therapeutic efficacy.

## 1. Introduction

Tumors are characterized by uneven vascular perfusion. The outer-most regions often have near normal blood flow while the inner regions can be avascular [1,2,3]. In addition, elevated interstitial fluid pressure and rigid extracellular matrix compromise intra-tumoral solute transport [4]. This leads to poor drug delivery to the under-perfused regions within the tumor and the eventual development of drug resistance [5,6,7]. Cell-based drug delivery can overcome these critical tissue barriers faced by synthetic nano drug delivery systems [8,9,10]. Because of their ability to respond to cytokine and chemokine gradients, various cell types including T cells [11], macrophages [12] and non-hematopoietic mesenchymal stem cells (MSCs) have been investigated for drug delivery [13]. Initial studies with these cell-based systems typically involved genetic modification of the cells to express anti-tumor peptides and proteins [14]. More recently, techniques that allow the functionalization of cells with nano drug delivery systems without affecting their viability or migratory phenotype have enabled the utilization of MSCs for small molecule drug delivery [15,16,17].

A critical parameter that influences the effectiveness of cell-based drug delivery is payload capacity. Previous studies have loaded nanoparticle-encapsulated drugs into cells through either simple endocytosis mediated uptake of nanoparticles or by covalent conjugation of nanoparticles to cell surface [15,16,18]. Rapid recycling and exocytosis of internalized nanoparticles [19] results in low loading capacities when relying on non-specific endocytosis of drug-loaded nanoparticles. Similarly, cells continuously internalize and recycle their outer membranes, which can result in lysosomal degradation of membrane-conjugated nanoparticles.

**Figure 1:**
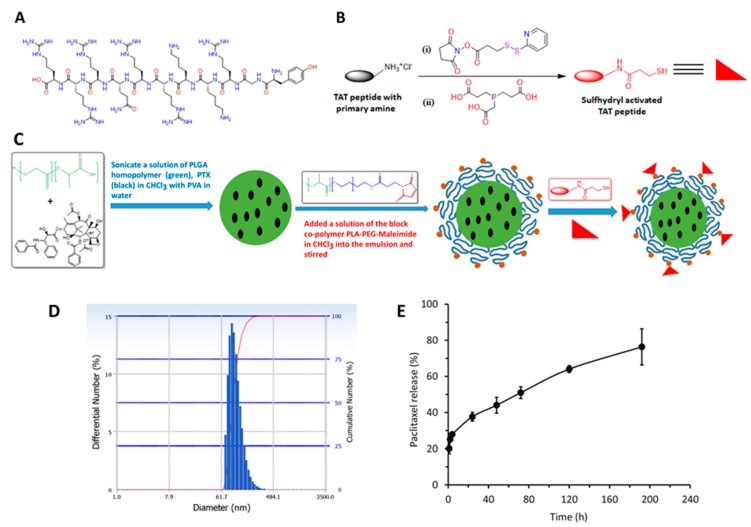
Preparation and characterization of PTX loaded TAT functionalized PLGA nanoparticles (TAT PTX NP). (**A**) Chemical structure of HIV-1 TAT peptide (47–57, YGRKKRRQRRR). (**B**) Synthetic scheme for the preparation of sulfhydryl activated TAT peptide using SPDP and TCEP. (**C**) Diagrammatic representation for the preparation of TAT PTX NP. (**D**) Particle size distribution of TAT PTX NP. (**E**) In vitro release profile of PTX from TAT PTX NP in cell culture medium supplemented with 10% (*w*/*v*) Captisol^®^ at 37 °C. Data shown is mean ± SD (*n* = 4).

In the current study, we evaluated nanoparticles functionalized with cell penetrating peptide (CPP) to enhance drug payload capacity of MSCs. CPPs are typically 5–30 amino acids long, and are characterized by their intrinsic ability to bypass lysosomes and enter cytoplasm through macropinocytosis [20]. Among the various CPPs identified so far, transactivator of transcription (TAT) peptide has been widely investigated to improve the intracellular delivery of various cargoes [21]. TAT peptide fragments are derived from the human immunodeficiency virus 1 protein containing 86–102 amino acid residues. The arginine-rich basic domain 48–60 (GRKKRRQRRRPPQ) is responsible for the transactivation [22]. TAT peptide (47 to 57 YGRKKRRQRRR, Figure 1A) has been successfully used to deliver biologically active antibodies [23], proteins [24], nucleic acids [25], small molecules [26], and nanocarriers [27,28] both in vitro and in vivo. TAT peptide and TAT conjugated systems are known to be internalized primarily through endocytic pathways [27,29,30]. Cellular internalization of TAT peptide is temperature-dependent, dynamin-1-independent, and is inhibited by drugs that block macropinocytosis [31]. However, some studies suggest that clathrin and caveolae-mediated endocytosis are likely also involved [32,33,34].

We hypothesized that surface functionalization of polymeric nanoparticles with TAT peptide will enable their improved internalization into and retention by MSCs, resulting in enhanced payload carrying capacity. We covalently conjugated TAT peptide to the surface of nanoparticles encapsulating paclitaxel (PTX) and used these nanoparticles to incorporate PTX in MSCs. Our studies show that these nano-engineered MSCs are effective in inhibiting tumor growth in a mouse orthotopic model of lung cancer.

## 2. Results

### 2.1. Synthesis of Thiolated TAT Peptide

To conjugate TAT peptide to nanoparticles having surface maleimide groups, the primary amine groups in the TAT peptide was converted into free thiol (-SH) using N-Succinimidyl 3-(2-pyridyldithio)propionate (SPDP) reagent and reducing agent tris(2-carboxyethyl)phosphine hydrochloride (TCEP). The detailed mechanism for the conversion is provided in the supporting information (Supplementary Figure S1). This reaction resulted in the conversion of one amine group (1.02 ± 0.12) per TAT peptide molecule into a free thiol as determined by thiol quantitation method.

### 2.2. Physicochemical Characterization of Nanoparticles

The hydrodynamic diameter (particle size) and zeta potential (surface charge) of nanoparticles were measured by dynamic light scattering (DLS) technique. Particle size of PTX loaded poly(DL-lactide-co-glycolide) (PLGA) nanoparticles (PTX NP), TAT functionalized PLGA nanoparticles (TAT NP), and PTX loaded TAT functionalized PLGA nanoparticles (TAT PTX NP) was 213 ± 6, 227± 18, and 225 ± 7 nm, respectively (Figure 1D). The surface charge of PTX NP, TAT NP, and TAT PTX NP was found to be −21 ± 1, −8.7 ± 2.5, and −15 ± 4 mV, respectively. PTX loading was in the range of 15–16% *w*/*w*. The efficiency of TAT peptide conjugation to NPs was 57 ± 4% (2.42 ± 0.14 µg/mg of NP).

The goal of the in vitro drug release study was to confirm that the incorporation of TAT peptide did not influence the drug release characteristics of nanoparticles. In order to maintain sink condition, PTX release study was conducted in cell culture medium supplemented with 10% (*w*/*v*) Captisol^®^. In vitro release of PTX from nanoparticles is shown in Figure 1E. An initial burst release of about 20% of the encapsulated PTX was observed in the first 1 h, followed by a steady release over the study period. The total PTX released over 8 days was ~76%. This was similar to that observed with non-functionalized PLGA nanoparticles in our previous studies [35], suggesting that TAT peptide functionalization did not affect the release profile of PTX from the nanoparticles. The initial burst release of PTX is primarily ascribed to the drug molecules present on or near the nanoparticle surface. However, it is noteworthy that the burst release took place within first 4 h, which is equivalent to optimal time for nano-engineering of MSCs. Thus, loosely bound PTX molecules are released during the nano-engineering process and nanoparticles loaded in the MSCs are in the sustained release phase.

### 2.3. Nanoparticles Uptake and Retention in MSCs

**Figure 2:**
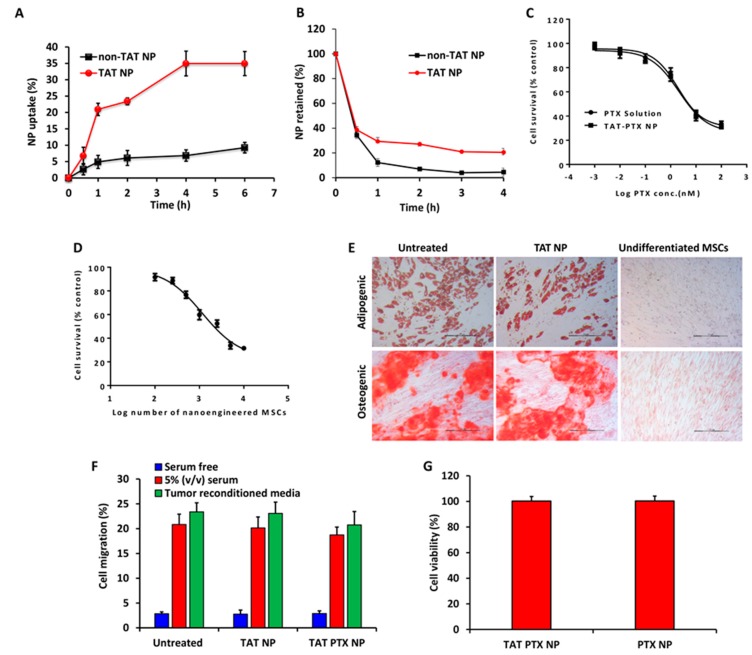
In vitro endocytosis (**A**) and exocytosis (**B**) of TAT-modified or non-TAT nanoparticles in MSCs. Cells were incubated with 100 µg/mL rhodamine-labeled PLGA nanoparticles. Data shown is mean ± SD, *n* = 4. (**C**,**D**) Cytotoxicity profiles of PTX solution, TAT PTX NP, and nano-engineered MSCs in A549 cells. Cells were treated with PTX solution or TAT PTX NP (**C**) or nano-engineered MSCs (**D**). MTS assay was performed after 72 h of treatment. Data shown is mean ± SD, *n* = 6. (**E**) Differentiation (adipogenic and osteogenic) potential of nano-engineered MSCs (MSCs engineered with TAT NP; TAT NP). Untreated MSCs and nano-engineered MSCs were grown in adipogenic and osteogenic differentiation media for 3 weeks. The cells were fixed and stained with oil red O or alizarin red to detect lipid vacuoles or calcium deposits, respectively. MSCs cultured in regular growth media were used as negative control. Scale bar: 200 µm. (**F**) The migratory potential of MSCs from a serum free media towards serum free, tumor reconditioned or 5% (*v*/*v*) serum containing media in a Transwell^®^ plate. Data shown is mean ± SD, *n* = 6. (**G**) Cell viability of nano-engineered MSCs. Data shown is mean ± SD, *n* = 5.

In order to determine the optimal incubation time to achieve the maximum nanoparticle loading in MSCs, we performed quantitative uptake studies. The uptake of TAT NP was 3-fold higher when compared to that of non-TAT NP (Figure 2A). These findings demonstrated the effectiveness of TAT peptide-functionalized nanoparticles in increasing the drug payload in MSCs. However, there was no significant difference in amount of TAT NP taken up at 4 or 6 h and hence 4 h was used as the optimal incubation time for subsequent nano-engineering processes. The amount of PTX loading in nano-engineered MSCs was quantified using HPLC. The average PTX content was found to be 16.1 ± 0.9 pg/cell in MSCs incubated with TAT PTX NP. As shown in Figure 2B, TAT-NP demonstrated a 5-fold increase in the % retained in the cells when compared to non-TAT-NP, suggesting that the TAT-functionalized nanoparticles not only enhanced payload capacity, but also improved the cellular retention of nanoparticles.

### 2.4. In Vitro Cytotoxicity Studies

The in vitro cytotoxicity potential of nano-engineered MSCs was determined in A549 cells using a standard MTS assay. PTX solution and TAT PTX NP were used as controls. IC50 values for nano-engineered MSCs, PTX solution, and TAT PTX NP were 1171 MSCs (Figure 2D) (equivalent to 22 nM PTX), 1.96 nM, and 2.10 nM (Figure 2C), respectively.

### 2.5. Characterization of Nano-Engineered MSCs

Nano-engineered MSCs were characterized for adipogenic and osteogenic differentiation potential. There were no apparent differences in the formation of neutral lipid vacuoles and calcium deposits, confirming that nano-engineered MSCs retain adipogenic and osteogenic differentiation potential, respectively (Figure 2E).

Next, we also confirmed the functional ability of the nano-engineered MSCs using an in vitro migration assay. As shown in Figure 2F, there was no significant change in the migration properties of nano-engineered MSCs when compared to untreated MSCs. In response to cytokines present in 5% serum media, 21% of untreated MSCs, 20% MSCs treated with TAT NP, and 19% MSCs treated with TAT PTX NP migrated to the lower chamber of the Transwell^®^ plate (Figure 2F). Similarly, 23% of untreated MSCs, 23% of MSCs treated with TAT NP, and 20% MSCs treated with TAT PTX NP migrated towards tumor reconditioned media (Figure 2F). These findings clearly demonstrated that loading MSCs with TAT PTX NP did not significantly affect the migratory ability of the MSCs.

### 2.6. Cell Viability of Nano-Engineered MSCs

The effect of TAT PTX NP on MSCs survival was evaluated by incubating MSCs with 100 µg/mL nanoparticles. There was no significant effect of TAT PTX NP on viability of MSCs (Figure 2G). Further, there was no difference in cell viability of TAT PTX NP and PTX NP treated MSCs, suggesting TAT conjugation did not alter the cytotoxic potential of nanoparticles.

### 2.7. Therapeutic Efficacy of Nano-Engineered MSCs in Orthotopic Lung Tumor Model

**Figure 3:**
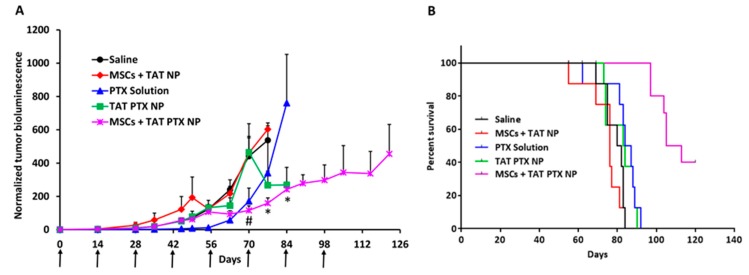
Nano-engineered MSCs were more effective in inhibiting orthotopic tumor growth and increased the overall survival of tumor bearing mice. (**A**) SCID beige mice bearing orthotopic A549 lung tumors were intravenously injected with Dulbecco’s phosphate buffered saline (Saline); 4 Million MSCs engineered with TAT NP (MSCs + TAT NP); PTX solution; TAT PTX NP; 4 Million MSCs engineered with TAT PTX NP (MSCs + TAT PTX NP). Plot of normalized bioluminescence readings (*n* = 10 for MSCs + TAT PTX NP and *n* = 8 for all other groups). Arrowheads indicate injection days. (*) Indicates significantly different (*p* < 0.05) from PTX solution; # indicates significantly different (*p* < 0.05) from TAT PTX NP. (**B**) Kaplan–Meier survival curves for the different treatment groups. Log rank test of MSCs + TAT PTX NP and control groups yields *p* < 0.0001 (*).

The anticancer efficacy of nano-engineered MSCs was evaluated in an orthotopic lung tumor model. Mice treated with MSCs + TAT PTX NP showed significant inhibition of tumor progression (*p* < 0.05) compared to other treatment groups (Figure 3A). Furthermore, MSCs + TAT PTX NP treated mice had significantly longer survival than those in control groups (log-rank test, *p* < 0.0001) (Figure 3B). Notably, the median survival of MSCs + TAT PTX NP treated mice was 109 days after treatment initiation, while the median survival of mice in control groups was in the range of 76–86 days.

### 2.8. Immunohistological Staining of Lung Tumors

**Figure 4:**
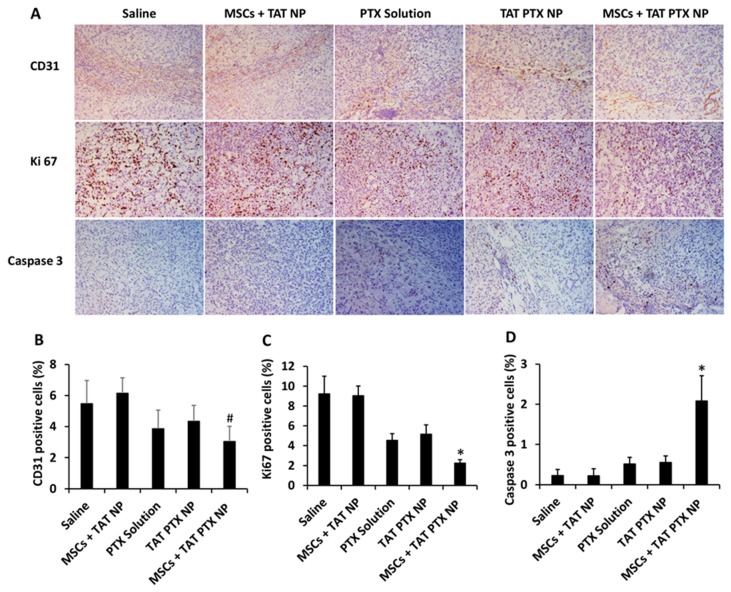
Immunohistological analysis of lung tumors collected from therapeutic efficacy study. (**A**) Lung tumors were stained for CD31 (angiogenesis marker), Ki-67 (proliferation marker), and caspase-3 (apoptosis marker). Images were taken at 20× magnification. Quantification of (**B**) CD31, (**C**) Ki67, and (**D**) cleaved caspase-3 staining. Data represented as mean ± SD, *n* = 9 images; * *p* < 0.05 compared with other treatment groups and # *p* < 0.05 compared with saline and MSCs + TAT NP.

In order to study the mechanism of improved anticancer efficacy with nano-engineered MSCs, lung tumor sections were stained for CD31, Ki-67, and cleaved caspase 3 (Figure 4A). Lung tumors from the mice treated with nano-engineered MSCs showed significantly fewer angiogenic blood vessels compared to saline or MSCs + TAT NP treatment groups (*p* < 0.05). In addition, lung tumors from the mice treated with nano-engineered MSCs showed significantly fewer proliferating cells (Ki-67 positive cells) compared to other treatment groups (*p* < 0.05). Densities of the cleaved caspase 3 positive apoptotic cells in tumors from nano-engineered MSC treatment group was significantly higher (panel 3 of Figure 4A) than those in other groups (*p* < 0.05).

### 2.9. Toxicology Assessment of Nano-Engineered MSCs

To examine the possible side effects of nano-engineered MSCs, we evaluated various biochemical and hematological parameters in healthy mice. We did not observe any signs of distress or significant differences in the body weights of the mice in different groups. Levels of aspartate aminotransferase (AST), alanine aminotransferase (ALT), gamma-glutamyl transferase (GGT), alkaline phosphatase (ALP), total protein (TP), albumin (ALB), globulin (GLOB), albumin/globulin (A/G) ratio, and total bilirubin (TBIL) in serum collected from treated mice were not different than those in the saline group (Table 1). Treatments with MSCs + TAT NP and MSCs + TAT PTX NP resulted in about 30% to 60% increase in platelet count (Table 2). Previous studies have shown that MSCs can increase platelet count through induction of interlukein-10 (IL-10) and TGF-β [36]. None of the other hematological parameters were affected by the treatments (Table 2, Table S1).

## 3. Discussion

Current approaches to tumor targeted drug delivery rely on passive accumulation of the drug carrier in the tumor through the ‘enhanced permeation and retention’ (EPR) effect, followed by internalization into tumor cells through either non-specific endocytosis or specific receptor-mediated endocytosis. However, the leaky tumor vasculature and dysfunctional lymphatics that allow for enhanced permeation of nano delivery systems also result in elevated interstitial fluid pressure that inhibits convective transport within the tumor microenvironment. In addition, tumors have a dense extracellular matrix that hinders diffusion. These physiologic and anatomic barriers constrain the extent of drug distribution within the tumor and limit the overall therapeutic effectiveness of synthetic delivery systems.

Recent studies have shown that cell-based drug carriers such as MSCs can infiltrate tumor tissue more uniformly, and thus improve the intra-tumoral distribution of the therapeutic payload. MSCs have been shown to actively traffic to both primary tumors and metastases, in response to inflammatory signals secreted by neutrophils and macrophages infiltrating the tumor, which enables the possibility of true active targeting of anticancer agents to the tumor tissue [13]. However, it is difficult to load small molecules in cells because of their diffusional clearance out of the cells. In addition, overexpression of drug efflux transporters such as P-glycoprotein in MSCs limits the loading of anticancer drugs, many of which are substrates for efflux transporters [37]. Several attempts have been made to load MSCs with anticancer therapeutics using polymeric nanoparticles [17,35,38], micelles [39], liposomes [40], carbon nanotubes [15], and dendrimers [41]. A key limitation with these methods is the limited drug loading efficiency [35,37]. For example, Zhang et al. investigated MSCs to deliver doxorubicin-polymer conjugates for glioma therapy [41]. However, the doxorubicin content in MSCs (5.81 ± 0.27 pg/cell) was found to be inadequate to meet the effective dose needed for systemic administration based on the maximum number of cells that could be injected. In our previous study, PTX loaded PLGA nanoparticles were used to load MSCs, but exocytosis of PLGA nanoparticles from MSCs reduced the amount of nanoparticle- and PTX payload that can could be loaded in MSCs (4.7 pg/cell) [35]. We hypothesized that this limitation can be overcome by increasing the cellular uptake and retention of nanoparticles. It has been previously shown that TAT peptide can be used to enhance the intracellular delivery of diverse bioactive molecules [23,24,25,26,27,28]. Attachment of TAT peptide to the surface of a drug carrier was shown to enhance the cellular uptake of the carrier [42]. Feiner-Gracia et al. also demonstrated that TAT functionalized PLGA nanoparticles were efficient in crossing plasma membrane and releasing their cargo inside the cells [43]. The increased uptake of TAT functionalized nanoparticles was attributed to the cationic nature of peptide, which was responsible for strong electrostatic interactions with anionic cell membrane, resulting in permeabilization and hence enhanced penetration inside the cells. Based on these findings, PLGA nanoparticles surface functionalized with TAT peptide were used to increase the drug loading capacity of MSCs. Our results further demonstrated that TAT functionalization improved drug loading in MSCs by ~3.4-fold compared to that with non-functionalized nanoparticles by increasing the uptake as well as retention of nanoparticles inside MSCs.

Although our aim was to generate nano-engineered MSCs with high drug loading, it was also critical to ensure that nano-engineering process did not alter MSCs phenotype or their viability. Our studies demonstrate that MSCs engineered with TAT NP retained their capacity to undergo osteogenic and adipogenic differentiation. Similarly, loading MSCs with TAT PTX NP did not affect their migration and tumor tropism, a property critical for their use in tumor-targeted drug delivery. Additionally, the viability of nano-engineered MSCs is essential for their tumor homing and sustained release of drugs at the tumor site. TAT conjugated nanoparticles did not result in toxicity when incubated with MSCs at the concentrations required for nano-engineering process. These results confirmed that TAT PTX NP loading had no negative impact on the phenotype or viability of MSCs.

Systemic injection of high number of MSCs can cause micro embolism, which could further lead to vascular obstruction, stroke and/or death. To evaluate the maximum number of MSCs that can be administered intravenously without causing adverse effects, we dosed 2, 4, and 6 million MSCs/mouse. This preliminary study showed that a bolus dose of 2 or 4 million MSCs did not cause any gross toxicity or adverse effects over a one-week observation period. Based on the results of this study, we selected a dose of 4 million nano-engineered MSCs/mouse (equivalent to 3.2 mg per kg BW PTX). Despite significantly lower total dose of PTX (25.6 mg/kg total dose for MSCs + TAT PTX NP Vs 120 mg/kg total dose for free drug), nano-engineered MSCs resulted in significantly greater tumor inhibition compared to free drug. Importantly, the mean survival of mice treated with nano-engineered MSCs (109 days for MSCs + TAT PTX NP) was higher than that for the mice treated with PTX solution (86 days). Further, PTX has been shown to cause several dose-dependent toxicities such as leukopenia, neutropenia, and abnormalities in liver enzymes, including ALP, ALT and bilirubin [44,45]. Our previous studies also demonstrated that treatment with PTX solution (administered at 40 mg/kg on day 0, 4, and 8) led to decrease in both WBC and RBC count as well as abnormalities in liver enzyme induction [16]. However, in our current studies we did not observe any of these toxicities.

Finally, immunohistochemical studies confirmed that PTX delivered using nano-engineered MSCs resulted in greater inhibition of angiogenesis, decreased tumor cell proliferation and increased apoptosis, all of which point to improved tumor delivery of the drug with MSCs nano-engineered using TAT PTX NP.

## 4. Materials and Methods

### 4.1. Materials

TAT peptide, TCEP, polyvinyl alcohol (PVA), alizarin red and oil red O were obtained from Sigma (St. Louis, MO, USA). SPDP was purchased from Biovision (Milpitas, CA, USA). Amplite™ fluorimetric total thiol quantitation assay kit was purchased from AAT Bioquest, Inc. (Sunnyvale, CA, USA). PTX was purchased from TCI America, Portland, OR, USA. FITC labeled TAT peptide (FITC-LC-YGRKKRRQRRR-NH2) was purchased from AnaSpec (Fremont, CA, USA). Ester-terminated 50:50 poly (DL-lactide-co-glycolide) (inherent viscosity: 0.55–0.75 dL/g) was purchased from Lactel Absorbable Polymers (Birmingham, AL, USA). Poly (L-lactide)-b-polyethylene glycol-maleimide and poly (lactide-co-glycolide)-rhodamine B (lactide to glycolide ratio of 50:50, rhodamine B endcap, Mn 10,000–30,000 Da) were purchased from PolySciTech (West Lafayette, IN, USA). Fetal bovine serum (FBS) and penicillin/streptomycin were procured from Bioexpress (Kaysville, UT, USA). RPMI 1640, Dulbecco’s phosphate buffered saline (DPBS), and trypsin-EDTA solutions were purchased from Invitrogen Corporation (Carlsbad, CA, USA). Mesenchymal stem cell media (MSCM) and human MSCs were obtained from ScienCell Research Laboratories (Carlsbad, CA, USA). A549-luc cell line was purchased from Caliper Life sciences (Waltham, MA, USA). D-Luciferin potassium salt was purchased from Gold Biotechnology (Saint Louis, MO, USA). Female Fox Chase SCID Beige mice (CB17.Cg-PrkdcscidLystbg-J/Crl) were purchased from Charles River Laboratories.

### 4.2. Synthesis of Sulfhydryl Activated TAT Peptide

Amine groups in the TAT peptide were converted into free thiols using the heterobifunctional cross-linker SPDP followed by treatment with TCEP (Figure 1B). TAT peptide (1 mg) was dissolved in PBS buffer. A 2-fold molar excess of SPDP was added to TAT peptide solution and incubated on a rotating platform for 2 h, followed by reduction with a 20-fold molar excess of TCEP for 2 h. The thiolated TAT peptide was purified using a polyacrylamide desalting column. The presence of thiol in TAT peptide was quantified using amplite™ fluorimetric total thiol quantitation assay kit (AAT Bioquest, Inc., Sunnyvale, CA, USA). The thiolated TAT peptide was subsequently used to functionalize nanoparticles containing surface maleimide groups.

### 4.3. Preparation of TAT-functionalized PLGA Nanoparticles

PTX loaded PLGA nanoparticles were prepared according to a previously described single emulsion-solvent evaporation technique (Figure 1C) [38]. In brief, PTX (7 mg) and PLGA (32 mg) were dissolved in 1 mL of chloroform and added to 8 mL of 2.5% *w*/*v* PVA solution. The mixture was sonicated using a probe sonicator set at an output of 18–21 W for 5 min (Sonicator XL, Misonix, NY, USA). The block co-polymer poly(L-lactide)-b-polyethylene glycol-maleimide (PLA-PEG-Mal; 8 mg) was dissolved in 0.2 mL chloroform and added to the emulsion with continuous stirring. The emulsion was further stirred overnight under ambient conditions, followed by 1 h stirring under vacuum to completely remove chloroform. The resulting nanoparticles were washed by ultracentrifugation at 35,000 rpm for 35 min at 4 °C (Optima XPN-80 Ultracentrifuge, Rotor type: 50.2 Ti, Beckman Coulter) followed by resuspension in deionized water three times. After the final wash, the nanoparticle pellet was dispersed in deionized water and centrifuged at 1000 rpm for 10 min. The supernatant was then reacted with thiolated TAT peptide. The resulting dispersion was stirred overnight at 4 °C. TAT peptide functionalized nanoparticles (TAT PTX NP) were then centrifuged to remove unreacted TAT peptide, and lyophilized (Labconco, FreeZone4.5). Blank nanoparticle formulation without the drug (TAT NP) was synthesized similarly. Drug-free, rhodamine-labeled nanoparticles were formulated by adding 5 mg PLGA-rhodamine B to 27 mg PLGA (total 32 mg polymer).

### 4.4. Characterization of Nanoparticles

Delsa Nano C particle analyzer (Beckman Coulter, California, USA) was used to determine the hydrodynamic diameter and zeta potential (surface charge). Nanoparticles were dispersed in deionized water (0.1 mg/mL) using probe sonication (18–21 W for one min). Analysis was performed at 25 °C and a scattering angle of 165°.

To determine PTX loading, nanoparticles were dispersed in methanol (1 mg/mL) and the drug was extracted overnight using a rotary extractor at room temperature. Nanoparticles were separated from free PTX by centrifugation at 13,000 rpm for 30 min, and PTX concentration of the methanolic extract was analyzed using HPLC [46].

To determine the extent of TAT peptide conjugation, nanoparticles were prepared with FITC-labeled TAT peptide and fluorescence spectroscopy was used to measure TAT associated fluorescence. Briefly, 1 mg of FITC-TAT nanoparticles was dispersed in deionized water and fluorescence intensity (λex: 493 and λem: 522 nm) was recorded using a SpectraMax i3x multi-mode microplate reader (Molecular Devices, LLC, CA, USA). Nanoparticles formulated without FITC-TAT were used as blank control. The amount of TAT peptide conjugated to nanoparticles was quantified using the standard curve of FITC-TAT solutions in deionized water (0.25–32 µg/mL).

The release of PTX from TAT PTX NPs was determined in MSC culture medium supplemented with 10% *w*/*v* Captisol^®^ (Cydex Pharmaceuticals, Lawrence, KS). Aliquots of nanoparticle dispersion (0.5 mL, 0.1 mg/mL) were kept in an incubator shaker (37 °C; 100 rpm). At each time point (1 h, 2 h, 4 h, 1 day, 2 days, 3 days, 5 days, and 8 days), samples (*n* = 4) were centrifuged at 13,000 rpm for 15 min. The supernatant (0.45 mL) was collected and analyzed directly for PTX content by HPLC as described above.

### 4.5. Cell Culture

Human MSCs were cultured in human MSC complete medium (ScienCell Research Laboratories, Carlsbad, CA, USA). A549-luc cells were cultured in RPMI 1640 medium containing 10% FBS, penicillin (100 IU/mL) and streptomycin (100 μg/mL). All the cells were cultured at 37  °C in a humidified incubator containing 5% CO_2_ and 95% air and monitored regularly for morphology and growth characteristics.

### 4.6. Nanoparticle Uptake and Retention in MSCs

For uptake studies, nanoparticles were fabricated using PLGA polymer that was covalently labeled with rhodamine. MSCs were plated onto a 24-well plate at a density of 1 × 10^4^ cells/well in 0.5 mL of MSC growth medium. Next day, the growth medium was removed, and cells were incubated with nanoparticle dispersion in culture medium (100 µg/mL) for 0.5 h, 1 h, 2 h, 4 h, and 6 h at 37 °C. At each time point, a group of wells were washed 3 times with DPBS and the cells in those wells were lysed in 300 μL of DPBS by subjecting them to 3 freeze thaw cycles. The amount of nanoparticles in the cell lysates was determined by monitoring rhodamine fluorescence using an IVIS spectrum imaging system (Caliper Life Sciences, λex: 535 nm and λem: 580 nm). For the retention study, cells were incubated with nanoparticles for 4 h followed by two washes with DPBS (this was designated as the 0 h time point). Cells were further incubated with fresh culture medium at 37 °C. At various time points over 4 h, cells were washed with DPBS, lysed and the amount of nanoparticles in the cell lysate was determined. Data was represents % of nanoparticles retained insides cells relative to the 0 h time point.

### 4.7. Preparation of Nano-Engineered MSCs

MSCs in suspension (2 × 10^5^ cells/mL) were incubated with nanoparticle dispersion in cell culture medium (100 µg/mL) in a rotating shaker at 37 °C. After 4 h of incubation, the cell suspension was washed thrice by centrifugation at 1000 rpm for 5 min (Allegra X-30R Centrifuge, Rotor type: SX4400, Beckman Coulter) followed by resuspension in DPBS to remove uninternalized nanoparticles. The final cell pellet was resuspended in DPBS for further studies.

### 4.8. In Vitro Migration Potential of Nano-Engineered MSCs

In vitro migration potential of nano-engineered MSCs was evaluated using a 96-Transwell^®^ plate. MSCs were serum starved for 24 h prior to the migration study. To initiate the study, 5 ×10^3^ untreated or nano-engineered MSCs in 50 µL serum-free medium were added to the top well of a 96-well Transwell^®^ plate separated by an 8.0 μm pore size PET membrane (Corning Life Sciences, Lowell, MA, USA). Bottom wells were filled with 200 µL of 5% (*v*/*v*) serum containing, serum-free, or tumor-reconditioned media. Tumor reconditioned media was generated by incubating A549 cells with 5% (*v*/*v*) serum containing media for 24 h. After incubating at 37 °C for 24 h, both top and bottom wells were washed with DPBS and 150 µL of calcein AM solution (1.2 μg/mL) in cell dissociation media was added to the bottom well followed by 1 h incubation in the dark at 37 °C. The cell suspension was transferred to a black-walled 96-well plate and the fluorescence intensities were recorded at excitation and emission wavelengths of 485 nm and 520 nm, respectively. The number of migrated cells was quantified using standard curves constructed using untreated and nano-engineered MSCs stained with calcein AM.

### 4.9. Effect of Nano-Engineering on MSC Viability

MSCs were seeded in a 24 well plate at a density of 1 × 10^4^ cells/well. Next day, cells were treated with 100 μg/mL nanoparticles at 37 °C. After 4 h incubation, nanoparticle dispersion was removed, cells were washed three times with DPBS, and the cell viability was determined by MTS assay. MSCs grown in cell culture medium was used as a control and percent cell survival was calculated using the following equation:

(1) $$Cell\;survival\;\left(\%\right)\;=\;\frac{\left(Absorbance\;of\;treated\;cells-background\right)}{\left(Absorbance\;of\;untreated\;cells-background\right)}\times 100$$

### 4.10. Differentiation Potential of Nano-Engineered MSCs

Nano-engineered MSCs were seeded at a density of ~4 × 10^4^ cells/well in a 24-well plate, and then incubated with adipogenic or osteogenic differentiation media (StemPro Osteogenesis or Adipogenesis Differentiation Kits, Life Technologies, Carlsbad, CA, USA) for 3 weeks. The medium was replaced every 3–4 days. The untreated MSCs incubated with differentiation media and those incubated with regular culture media were used as positive and negative controls, respectively. After a 3-week incubation period, cells were fixed with 4% formalin and stained with alizarin red or oil red O to visualize osteogenic and adipogenic differentiation, respectively. Images were captured using a light microscope at 20× magnification.

### 4.11. In Vitro Cell Growth Inhibition Studies

The cytotoxicity of nano-engineered MSCs against A549 (human lung adenocarcinoma) cells was evaluated using MTS assay. A549 cells were seeded at a density of ~2 × 10^4^ cells per well in 600 µL of RPMI 1640 medium in the bottom chamber of a 24 well Transwell^®^ plate. Nano-engineered MSCs at different cell densities (in 100 µL medium) were added to the top well of the Transwell^®^ plate separated by a 0.2 µm pore size PET membrane. PTX solution and TAT PTX NP were used as positive controls. After 3 days of incubation, A549 cell viability was determined by MTS assay. A549 cells grown in RPMI 1640 medium was used as a control, and the percentage of cell survival was calculated using Equation 1.

### 4.12. Therapeutic Efficacy of Nano-Engineered MSCs in Orthotopic Lung Tumor Model

All experiments involving animals were approved by the Institutional Animal Care and Use Committee (IACUC) of the University of Minnesota (Animal protocol No.: 1605-33821A, approval date: 25 July 2016). Female Fox Chase SCID Beige mice (CB17.Cg-PrkdcscidLystbg-J/Crl), 6–8 weeks old (each weighing 18–21 g), were injected intravenously with 1 × 10^6^ A549-luc cells dispersed in 200 µL DPBS. Tumor growth in the lungs was monitored by bioluminescence imaging. Mice were injected intraperitoneally with 150 mg/kg of D-luciferin potassium salt solution prior to imaging on an IVIS spectrum in vivo imaging system (Caliper Life Sciences). When tumor bioluminescence reached ~5 × 10^5^ photons/sec, mice were randomly assigned to five groups and were treated with intravenous injection of DPBS (200 µL at every 14 days, ‘Saline’; *n* = 8), PTX solution (administered at 40 mg/kg on day 0, 4, and 8; ‘PTX solution’; *n* = 8), TAT PTX NP (equivalent to 3.2 mg/kg PTX every 2 weeks; ‘TAT PTX NP’; *n* = 8), MSCs loaded with TAT NP (4 × 10^6^ every 2 weeks, ‘MSCs + TAT NP’, *n* = 8), or MSCs loaded with TAT PTX NP (4 × 10^6^ MSCs equivalent to 3.2 mg/kg PTX every 2 weeks, ‘MSCs + TAT PTX NP’, *n* = 10). Dosing regimen for PTX solution was selected based on our previously published results [16]. Tumor growth was monitored by imaging tumor bioluminescence twice weekly initially and then once a week. Animals were euthanized using CO_2_ when they showed signs of stress such as loss of appetite, weight loss, and/or ruffled hair. Tumor-bearing lungs were collected at the end of the study and processed for immunohistochemistry.

### 4.13. Toxicology Assessment of Intravenously Administered Nano-Engineered MSCs

Healthy SCID beige mice were administered with DPBS (control, *n* = 8), TAT PTX NP (administered at 3.2 mg per kg BW PTX on day 0 and 14, *n* = 8); MSCs + TAT NP (4 × 10^6^ cells on day 0 and 14, *n* = 8); MSCs + TAT PTX NP (4 × 10^6^ cells, equivalent to 3.2 mg per kg BW PTX on day 0 and 14, *n* = 8). For complete blood count (CBC), ~250 µL blood per mouse was collected from four mice in each group into EDTA tubes and gently mixed to prevent clotting. For liver function test, ~300 µL blood was collected from remaining four mice in each group into heparin-coated tubes. All the samples were analyzed by Charles River Clinical Pathology Services (Shrewsbury, MA, USA).

### 4.14. Immunohistological Staining of Lung Tumors

Lung tumors from the therapeutic study were fixed in 4% *w*/*v* formaldehyde solution for 24 h and subsequently transferred to 70% (*v*/*v*) ethanol. Tissue samples were embedded in paraffin and sectioned into 4 μm-thick slices. The sections were deparaffinized and stained for cleaved caspase-3, Ki67, and CD31. Both cleaved-caspase 3 (Cell Signaling Technology, Danvers, MA, USA) and Ki-67 clone SP-6 (Biocare Medical, Concord, CA, USA) staining used a 1:100 antibody concentration followed by Envision Rabbit Horseradish Peroxidase (HRP) detection system (Dako, Carpinteria, CA, USA). CD31 assay used a 1:1200 antibody concentration (Santa Cruz Biotechnology, Santa Cruz, CA, USA) followed by Goat-on-Rodent HRP polymer system (Biocare Medical). All the sections were developed using DAB chromogen (Dako) and counterstained with Mayer’s Hematoxylin (Dako). The relative staining for each target was analyzed by Image J software (https://imagej.nih.gov/ij/download.html) to determine the fraction of positive stain per unit tissue area.

### 4.15. Statistical Analysis

The statistical significance of observed differences between groups was determined by one-way ANOVA, followed by Bonferroni-Holm post-hoc analysis for comparison between individual groups. Log-rank test was conducted to compare the survival distribution of different treatment groups. A probability level of *p* < 0.05 was considered significant.

## 5. Conclusions

In summary, we demonstrated significantly improved drug loading in MSCs by using TAT functionalized nanoparticles. These nano-engineered MSCs retained their osteogenic and adipogenic differentiation properties and tumor-tropism. Moreover, nano-engineered MSCs were effective in inhibiting tumor growth and increasing the overall survival in a mouse orthotopic lung tumor model.

## Figures and Tables

**Table 1:** Effect of different treatments on liver function test. Data represents mean ± SD (*n* = 4).

Parameters	Saline		MSCs + TAT NP		TAT PTX NP		MSCs + TAT PTX NP	
	Day 7	Day 18	Day 7	Day 18	Day 7	Day 18	Day 7	Day 18
ALT (U/L)	33.0 ± 7.0	37.0 ± 5.3	33.5 ± 4.7	36.0 ± 5.4	32.5 ± 4.5	33.5 ± 5.5	35.8 ± 6.4	40.0 ± 4.4
AST (U/L)	80.7 ± 9.7	87.3 ± 13.1	85.8 ± 11.5	90.0 ± 10.8	85.7 ± 7.7	90.3 ± 10.0	84.8 ± 11.7	91.5 ± 5.8
GGT(U/L)	1.0 ± 0.0	1.0 ± 0.0	1.0 ± 0.0	1.0 ± 0.0	1.0 ± 0.0	1.0 ± 0.0	1.0 ± 0.0	1.0 ± 0.0
ALP (U/L)	94.3 ± 5.9	100.7 ± 7.1	90.3 ± 4.9	96.8 ± 11.0	95.5 ± 4.2	98.0 ± 16.5	95.0 ± 6.5	100.8 ± 10.3
TP (g/dL)	5.8 ± 0.2	5.4 ± 0.3	5.8 ± 0.3	5.9 ± 0.3	5.7 ± 0.2	5.5 ± 0.1	5.8 ± 0.2	5.3 ± 0.1
ALB (g/dL)	3.5 ± 0.1	3.2 ± 0.1	3.5 ± 0.2	3.4 ± 0.2	3.5 ± 0.1	3.4 ± 0.1	3.5 ± 0.1	3.4 ± 0.1
GLOB (g/dL)	2.3 ± 0.1	1.9 ± 0.1	2.3 ± 0.2	2.5 ± 0.2	2.1 ± 0.2	2.0 ± 0.1	2.3 ± 0.1	2.4 ± 0.0
A/G	1.5 ± 0.1	1.7 ± 0.1	1.6 ± 0.1	1.4 ± 0.1	1.7 ± 0.1	1.7 ± 0.1	1.5 ± 0.1	1.4 ± 0.0
TBIL (mg/dL)	0.3 ± 0.1	0.4 ± 0.1	0.3 ± 0.0	0.2 ± 0.0	0.3 ± 0.1	0.3 ± 0.0	0.2 ± 0.0	0.3 ± 0.1

Abbreviations: ALT-alanine aminotransferase; AST-aspartate aminotransferase; GGT-gamma-glutamyl transferase; ALP-alkaline phosphatase; TP-total protein; ALB-albumin; GLOB-globulin; A/G-albumin/globulin ratio; TBIL-total bilirubin.

**Table 2:** Effect of different treatments on complete blood count. Data represents mean ± SD (*n* = 4).

Parameters	Saline		MSCs + TAT NP		TAT PTX NP		MSCs + TAT PTX NP	
	Day 7	Day 18	Day 7	Day 18	Day 7	Day 18	Day 7	Day 18
WBC (×10^3^ cells/µL)	5.0 ± 1.4	6.3 ± 0.5	4.9 ± 0.8	5.0 ± 0.6	5.3 ± 0.6	4.4 ± 0.2	5.6 ± 1.2	4.7 ± 0.7
RBC (×10^6^ cells/µL)	9.8 ± 0.3	9.7 ± 0.3	9.8 ± 0.2	9.5 ± 0.3	9.8 ± 0.1	9.1 ± 0.3	9.7 ± 0.2	9.1 ± 0.1
HGB (g/dL)	15.4 ± 0.4	15.5 ± 0.6	15.3 ± 0.3	15.0 ± 0.5	15.6 ± 0.2	14.8 ± 0.3	15.1 ± 0.3	14.3 ± 0.3
HCT (%)	50.9 ± 1.1	49.2 ± 1.7	50.2 ± 1.0	46.5 ± 1.6	51.4 ± 0.7	46.1 ± 1.5	49.5 ± 0.9	45.6 ± 0.9
PLT (×10^3^ cells/µL)	840 ± 26	977 ± 50	1106 ± 224	1248 ± 131	914 ± 60	1060 ± 103	1349 ± 238	1234 ± 147

Abbreviations: WBC-white blood cell; RBC-red blood cell; HGB-hemoglobin concentration; HCT-hematocrit; PLT-platelets.

## Supplementary Materials

The following are available online at https://www.mdpi.com/2072-6694/11/4/491/s1, Figure S1: Preparation of TAT functionalized PLGA nanoparticles, Table S1: Effects of different treatments on complete blood count.
