# Supplementary material for: Improving Payload Capacity and Anti-Tumor Efficacy of Mesenchymal Stem Cells Using TAT Peptide Functionalized Polymeric Nanoparticles
Source: Cancers (Basel). 2019 Apr 6;11(4):491. doi: 10.3390/cancers11040491 (PMC6521160; doi:10.3390/cancers11040491)
Supplement: Supplementary file 1 [file cancers-11-00491-s001.pdf]

Supplementary Materials: Improving Payload Capacity and Anti-Tumor Efficacy of Mesenchymal Stem Cells Using TAT Peptide Functionalized Polymeric Nanoparticles

Gopikrishna Moku, Buddhadev Layek, Lana Trautman, Samuel Putnam, Jayanth Panyam, and Swayam Prabha

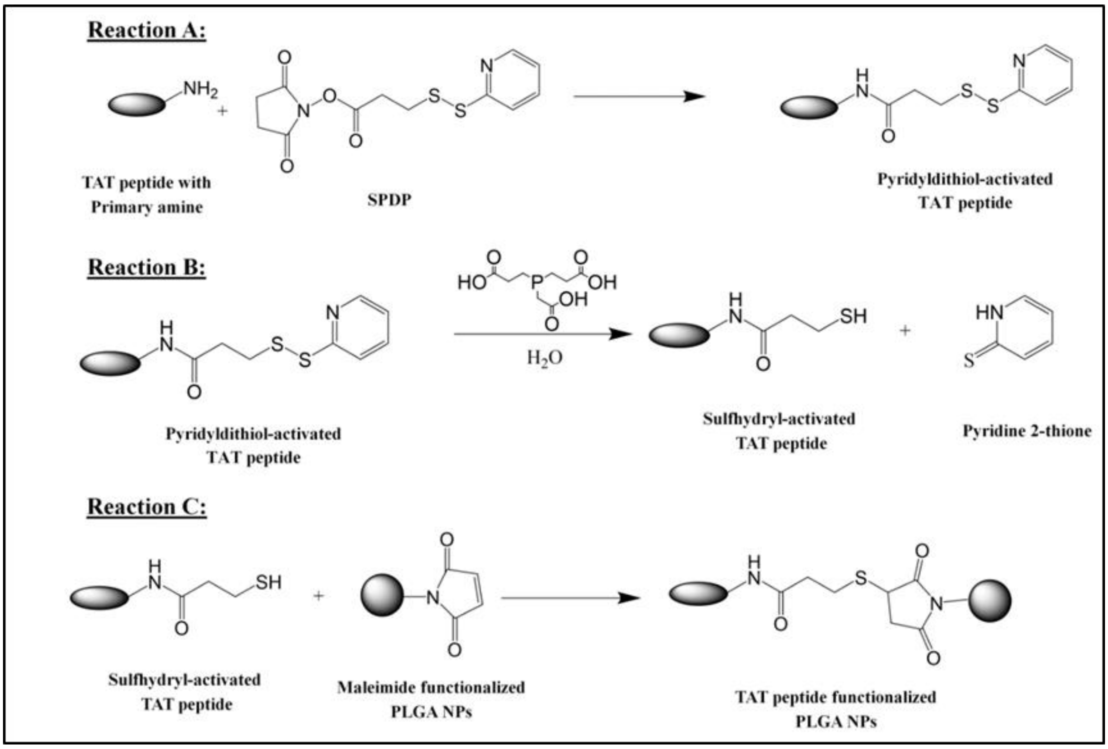

Figure S1. Preparation of TAT functionalized PLGA nanoparticles.

Table S1. Effects of different treatments on complete blood count. Data represents mean ± SD (n = 4).

Parameters	Saline		MSCs + TAT NP		TAT PTX NP		MSCs + TAT PTX NP	
	Day 7	Day 18	Day 7	Day 18	Day 7	Day 18	Day 7	Day 18
WBC (×10³ cells/μL)	5.0 ± 1.4	6.3 ± 0.5	4.9 ± 0.8	5.0 ± 0.6	5.3 ± 0.6	4.4 ± 0.2	5.6 ± 1.2	4.7 ± 0.7
NE (%)	16.3 ± 2.2	16.1 ± 3.3	14.1 ± 2.8	16.2 ± 5.2	13.4 ± 1.2	10.9 ± 4.1	11.7 ± 0.2	9.0 ± 1.7
LY (%)	77.7 ± 2.2	78.5 ± 3.1	80.7 ± 2.3	81.3 ± 5.9	81.5 ± 1.8	87.0 ± 6.0	82.3 ± 2.1	88.2 ± 3.2
MO (%)	1.5 ± 0.3	2.1 ± 0.3	1.7 ± 0.4	0.3 ± 0.6	1.5 ± 0.2	1.0 ± 0.7	1.7 ± 0.3	0.7 ± 0.5
EO (%)	2.9 ± 0.2	2.2 ± 1.1	2.3 ± 0.9	1.9 ± 1.7	2.1 ± 1.2	0.7 ± 1.0	3.1 ± 1.8	1.6 ± 1.0
BA (%)	0.3 ± 0.1	0.2 ± 0.1	0.2 ± 0.1	0.1 ± 0.1	0.3 ± 0.1	0.1 ± 0.1	0.2 ± 0.1	0.1 ± 0.1
LUC (%)	1.3 ± 0.2	0.9 ± 0.1	1.0 ± 0.2	0.2 ± 0.4	1.2 ± 0.3	0.3 ± 0.4	1.0 ± 0.3	0.4 ± 0.4
RBC (×10⁶ cells/μL)	9.8 ± 0.3	9.7 ± 0.3	9.8 ± 0.2	9.5 ± 0.3	9.8 ± 0.1	9.1 ± 0.3	9.7 ± 0.2	9.1 ± 0.1
HGB (g/dL)	15.4 ± 0.4	15.5 ± 0.6	15.3 ± 0.3	15.0 ± 0.5	15.6 ± 0.2	14.8 ± 0.3	15.1 ± 0.3	14.3 ± 0.3
HCT (%)	50.9 ± 1.1	49.2 ± 1.7	50.2 ± 1.0	46.5 ± 1.6	51.4 ± 0.7	46.1 ± 1.5	49.5 ± 0.9	45.6 ± 0.9
MCV (fL)	51.9 ± 0.5	51.0 ± 0.2	51.2 ± 0.3	49.1 ± 0.3	52.4 ± 0.8	50.5 ± 0.5	51.3 ± 0.6	49.9 ± 1.0
MCH (pg)	15.8 ± 0.2	16.0 ± 0.1	15.6 ± 0.2	15.8 ± 0.2	15.9 ± 0.3	16.2 ± 0.3	15.6 ± 0.2	15.7 ± 0.2
MCHC (g/dL)	30.3 ± 0.1	31.4 ± 0.1	30.5 ± 0.2	32.2 ± 0.5	30.3 ± 0.1	32.2 ± 0.5	30.6 ± 0.1	31.4 ± 1.0
RDW (%)	13.8 ± 0.5	14.7 ± 0.5	13.7 ± 0.3	13.9 ± 0.5	14.6 ± 0.4	14.8 ± 0.3	13.5 ± 0.9	14.3 ± 0.5
PLT (×10³ cells/μL)	840 ± 26	977 ± 50	1106 ± 224	1248 ± 131	914 ± 60	1060 ± 103	1349 ± 238	1234 ± 147
MPV (fL)	6.5 ± 0.2	7.8 ± 0.9	6.6 ± 0.4	6.8 ± 0.4	7.1 ± 0.5	7.1 ± 0.2	6.5 ± 0.3	6.6 ± 0.6

WBC: White Blood Cell; NE: Neutrophil; LY: Lymphocyte; MO: Monocyte; EO: Eosinophil; BA: Basophil; LUC: Large Unstained Cells; RBC: Red Blood Cell; HGB: Hemoglobin Concentration; HCT: Hematocrit; MCV: Mean Corpuscular

Volume; MCH: Mean Corpuscular Hemoglobin; MCHC: Mean Corpuscular Hemoglobin Concentration; RDW: RBC Distribution Width; PLT: Platelet count; MPV: Mean Platelet Volume.

© 2019 by the authors. Licensee MDPI, Basel, Switzerland. This article is an open access article distributed under the terms and conditions of the Creative Commons Attribution (CC BY) license (http://creativecommons.org/licenses/by/4.0/).
